# Characterization of the electronic structure and fate of doubly ionized carbon diselenide

**DOI:** 10.1038/s41598-025-90637-5

**Published:** 2025-02-24

**Authors:** Marco Parriani, Emelie Olsson, Veronica Daver Ideböhn, Måns Wallner, Richard J. Squibb, Gunnar Nyman, Stefano Falcinelli, John H. D. Eland, Majdi Hochlaf, Raimund Feifel

**Affiliations:** 1 Department of Civil and Environmental Engineering, University of Perugia, Via G. Duranti 93, 06125 Perugia, Italy; 2 Department of Physics, University of Gothenburg, Origovägen 6B, 412 58 Gothenburg, Sweden; 3 Department of Chemistry and Molecular Biology, University of Gothenburg, Box 462, 405 30 Gothenburg, Sweden; 4 Department of Chemistry, Physical and Theoretical Chemistry Laboratory, Oxford University, South Parks Road, Oxford, OX1 3QZ UK; 5 Université Gustave Eiffel, COSYS/IMSE, 5 Bd Descartes, 77454 Champs sur Marne, France

**Keywords:** Atomic and molecular interactions with photons, Chemical physics

## Abstract

Single photon double ionization of carbon diselenide (${\hbox {CSe}}_{2}$) has been investigated by means of multi-particle coincidence techniques. The interpretation of the experimental spectra is helped by post-Hartree-Fock computations at the Coupled Clusters and Multi-Reference Configuration-Interaction levels to determine the energetics and electronic state potentials of $\hbox {CSe}_2^{2+}$ and its fragments. The lowest experimental double ionization energy of ${\hbox {CSe}}_{2}$ has been found to be 24.68 ± 0.20 eV, reflecting the $\hbox { X} ^3\Sigma ^-_g$ ground state, and is in agreement with the theoretical vertical double ionization energy of 24.41 eV. Several fragmentation channels are reported including experimental appearance energies and kinetic energy releases in comparison to theoretical results on their characteristics. In particular, we identify several purely repulsive, Coulomb explosion fragmentation channels.

## Introduction

Investigations of the electronic structure of molecules have concentrated traditionally on their neutral forms, which are predominant in the terrestrial environment. When neutral molecules lose electrons, they form ions, species which are vital intermediates in many chemical reactions, particularly in atmospheric and extra-terrestrial contexts. Since the 1960s, spectra of molecular ions with one positive charge (monocations) have been measured directly by conventional photoelectron spectroscopy. Measurements of electron spectra of more highly ionized molecules and information on their fates had to wait much longer. They can now be carried out effectively by advanced multi-particle correlation spectroscopy techniques such as the TOF-PEPEPIPICO technique invented in the 2000s at Oxford University^1,2^. This type of experiment can reveal exotic, but nevertheless efficient pathways forming fundamental species like ${\hbox {O}}_2^{+}$ upon double ionisation of triatomic $\hbox {SO}_2$, which has the two oxygen atoms in terminal positions^3^.

Carbon diselenide (${\hbox {CSe}}_{2}$) is another triatomic with two equivalent atoms in terminal positions, and can be regarded as the selenium analogue of the highly-symmetric carbon disulfide (${\hbox {CS}}_{2}$) and carbon dioxide (${\hbox {CO}}_{2}$) molecules. Like ${\hbox {CS}}_{2}$, ${\hbox {CSe}}_{2}$ polymerises under high pressure. Among its chemical reactions relevant to technological applications, it is used as a precursor to tetraselenafulvalenes, which are central to the synthesis of organic conductors’ and organic superconductors^4^.

Even though ${\hbox {CSe}}_{2}$ was identified as an isolated species in the laboratory by Grimm and Metzger^5^ in the 1930s, our knowledge of its electronic structure and fragmentation processes in different states of charge and excitation is still limited. This is perhaps surprising, since already in the 1970s, single ionization of carbon diselenide had been investigated by photoelectron spectroscopy^6,7^ and mass spectroscopy^8–10^. Interestingly, despite the fact that ${\hbox {CSe}}_{2}$ has a linear symmetrical structure in its neutral ground state with the two Se atoms in terminal positions, the exotic $\text {Se}_2^{+}$ species is formed abundantly by singly ionizing ${\hbox {CSe}}_{2}$^8^ a few eV above the adiabatic ionisation energy of $\hbox {CSe}_2$, in addition to the conventional Se + $\hbox {CSe}^+$ and $\hbox {Se}^+$ + CSe product channels. This is in contrast to the dissociative photoionisation of its isovalent species, $\hbox {CO}_2$ and $\hbox {CS}_2$. Indeed, the latter are subject solely to carbon-chalcogen bond breaking upon single photon ionisation^11,12^. This is basically in line with the different ionisation energies of atomic O, S and Se and of the CX (X=O, S, Se) diatomics leading to different thermodynamical patterns of the corresponding dissociation limits.

Whether $\text {Se}_2^{+}$ is also formed in double ionization akin to the very recent finding of ${\hbox {O}}_2^{+}$ formation from dissociating $\text {SO}_2^{2+}$ (see Ref.^3^) and, similarly, of $\hbox {OS}^+$ formation from $\hbox {OCS}^{2+}$ (see Ref.^13^) is one of the open questions which we aim to shed light upon with the present combined experimental and theoretical work. Furthermore, we aim to investigate missing aspects of the electronic structure and associated nuclear dynamics of $\hbox {CSe}_2^{2+}$. In particular, we will investigate whether nuclear arrangements in a dense manifold of $\hbox {CSe}_2^{2+}$ electronic states are needed to explain the outcomes of the single photon double ionisation of $\hbox {CSe}_2$.

## Results and discussion

**Fig. 1:**
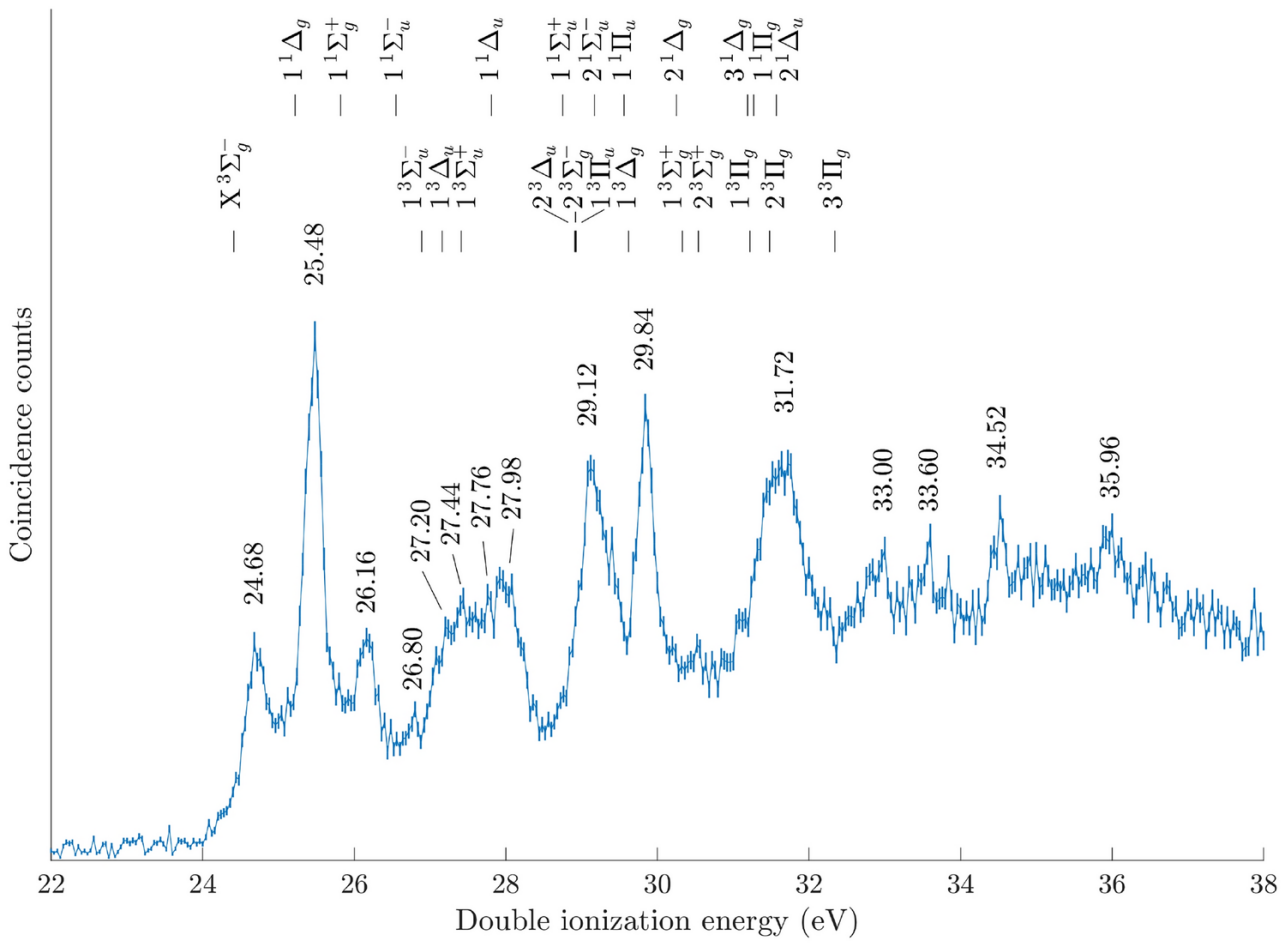
Valence double ionization electron spectrum of $\text {CSe}_2$ obtained at the He II$\alpha$ photon energy of 40.81 eV under electron-only conditions, reflecting several sharp features. Error bars represent the statistical uncertainty of the coincidence counts. Energy values given in the figure are based on the experimental results. For comparison, the theoretical vertical double ionization energies (VDIEs) are included as vertical lines.

Figure 1 shows the double ionization valence electron pair spectrum of $\text {CSe}_2$ obtained at the He II$\alpha$ photon energy of 40.81 eV using the electron-only configuration of our multi-particle correlation spectrometer set-up described in the “Methods” section below. The spectrum has an onset of about 24 eV ionization energy, and contains sharp features at 24.68 eV, 25.48 eV, 26.16 eV, 29.12 eV, 29.84 eV and 31.72 eV, as well as several less distinct features between 26.5 and 28 eV, and above 32.0 eV ionization energy, respectively. The adiabatic ionization energy of $\text {CSe}_2$ is computed as 24.16 eV at the MRCI/aug-cc-pVQZ-DK level. It corresponds to the onset of the first band in the spectrum of Fig. 1. The nominal resolution at these electron energies together with an uncertainty due to the procedure of calibrating the energy scale in absolute terms yield an uncertainty of the double ionization energy of ± 0.2 eV.

**Fig. 2:**
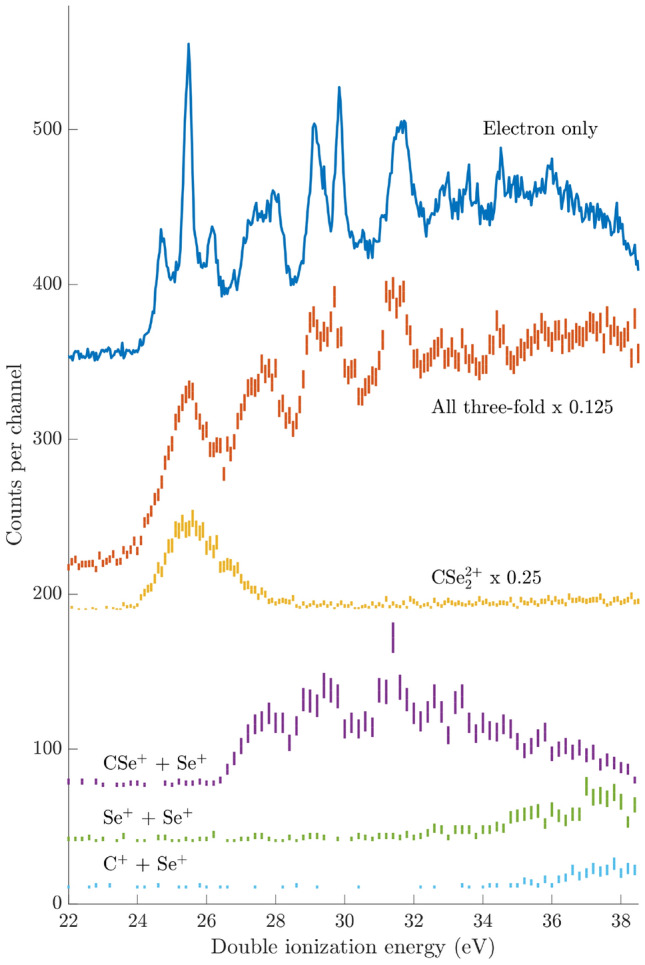
Valence double ionization electron spectra of ${\hbox {CSe}}_{2}$ obtained at the He II$\alpha$ photon energy of 40.81 eV under electron-ion conditions, with the breakdown products of double ionization labelled accordingly. For some of the fragment spectra, the intensities have been scaled by a factor stated in the figure. Error bars represent the statistical uncertainty of the coincidence counts. The uppermost spectrum is the electron-only spectrum from Fig. 1.

Figure 2 shows complementary valence double ionization electron pair spectra of ${\hbox {CSe}}_{2}$ measured at the He II$\alpha$ photon energy of 40.81 eV, where the more differential multi-electron-ion correlation set-up has been used to investigate the fate of doubly-ionized ${\hbox {CSe}}_{2}$ in respect to its ionic breakdown products. For comparison, the uppermost spectrum is the same as discussed in the context of Fig. 1, whereas the second spectrum from the top is based on three-fold coincidence events where all electron pairs were extracted in coincidence with the doubly-charged parent ion or the singly-charged cations $\text {CSe}^+$, $\text {Se}^+$, and $\text {C}^{+}$ from dissociating $\hbox {CSe}_2^{2+}$, respectively. As can be seen, the two spectra agree well, considering the difference in electron energy resolution for the electron-only and multi-electron-ion set-ups (see “Methods” section).

The lower panels of Fig. 2 present electron pair spectra extracted in coincidence with the doubly-charged parent ion (using three-fold events) and with ion pairs (using four-fold events) labelled accordingly $\text {CSe}^{+} + \text {Se}^{+}$, $\text {Se}^{+} +\text { Se}^+$, and $\text {Se}^{+} + \text {C}^+$, respectively. As can be seen, the first three lowest dicationic states starting at about 24.2 eV are primarily stable towards fragmentation. Starting at about 26.4 eV ionization energy, the formation of the $\text {CSe}^{+} + \text {Se}^{+}$ pair sets in. From about 32.2 eV on, the $\text {CSe}^{+} + \text {Se}^{+}$ channel competes with the formation of the ionic pair $\text {Se}^{+} +\text { Se}^+$, and from about 35 eV on also with the $\text {Se}^{+} + \text {C}^+$ fragmentation channel.

From the two-fold ion-ion coincidences it is possible to determine the kinetic energy release (KER) for the two most intense fragmentation channels. Estimates of the KER values are based on the FWHM width of the ion-ion coincidence peak and a computation of the electric field at the interaction point. The electrical field is estimated using a numerical replica of our setup in SIMION^14^, which provides the electrical field strength at different points in the interaction region. Since Se has several isotopes, the determination of the peak width is done by selecting the coincidence feature related to the most intense Se isotopes. For the $\text {CSe}^{+} + \text {Se}^{+}$ channel the KER from the peak width is 3.2 ± 0.2 eV, when including all electronic states, and for the $\text {Se}^{+} +\text { Se}^+$ channel the KER is 1.2 ± 0.2 eV.

To help the assignment of the features observed in Figs. 1 and 2, we performed ab initio computations on both the neutral and dicationic ${\hbox {CSe}}_{2}$ and its fragmentation channels. First, we optimized the geometry of the ground state of ${\hbox {CSe}}_{2}$ at the MRCI/aug-cc-pVQZ-DK and CCSD(T)/aug-cc-pVQZ-DK levels. Both computations lead to a centrosymmetric linear structure with a CSe distance of 1.70 Å, which agrees with the experimental value of 1.6919 Å, as determined by Bürger and Willner^15^. Also, the MRCI/aug-cc-pVQZ-DK harmonic frequencies of $\omega _1$ = 1316 $\hbox {cm}^{-1}$, $\omega _2$ = 315 $\hbox {cm}^{-1}$ and $\omega _3$ = 375 $\hbox {cm}^{-1}$ were computed. Assuming the reduction of these harmonic frequencies if taking into account the anharmonicity terms, we conclude to have a good agreement with the measured fundamentals (i.e. $\nu _1$ = 1254.30 $\hbox {cm}^{-1}$, $\nu _2$ = 302.89 $\hbox {cm}^{-1}$ and $\nu _3$ = 374.48 $\hbox {cm}^{-1})$ known in the literature^15^. This confirms the suitability of the MRCI/aug-cc-pVQZ-DK for studying the ${\hbox {CSe}}_{2}$ molecule and related neutral and ionic species, in particular for accounting for electron correlation and relativistic effects. Therefore, we started our computations dealing with $\text {CSe}_2^{2+}$ by mapping its lowest singlet and triplet potential energy surfaces (PES) at the MRCI/aug-cc-pVQZ-DK level. Close to the Franck–Condon region assessed from the ${\hbox {CSe}}_{2}$ equilibrium, we located a minimum structure in the singlet PES and a minimum structure in the triplet PES. Both structures are centrosymmetric linear with the $^3\Sigma _g^-$ and $^1\Delta _g$ symmetry species with CSe distances of $\sim$ 1.72 Å, slightly longer than that of neutral $\text {CSe}_2$ ($\hbox {X}^1\Sigma _g^+$). They are obtained after removal of two electrons from the outermost $\pi$ molecular orbital of ${\hbox {CSe}}_{2}$. This is associated with the weakening of the CSe bonds and results in low harmonic frequencies of ($\omega _1$ = 710 $\hbox {cm}^{-1}$, $\omega _2$ = 224 $\hbox {cm}^{-1}$, $\omega _3$ = 346 $\hbox {cm}^{-1}$, for $^3\Sigma _g^-$, and $\omega _1$ = 977 $\hbox {cm}^{-1}$, $\omega _2$ = 233 $\hbox {cm}^{-1}$, $\omega _3$ = 349 $\hbox {cm}^{-1}$, for $^1\Delta _g$). The triplet is lower in energy, and thus corresponds to the ground state of $\text {CSe}_2^{2+}$. These findings accord with the detection of $\text {CSe}_2^{2+}$ in Fig. 2.

For the assignment of the bands in the electron-only spectrum shown in Fig. 1, we computed the vertical double ionization energies (VDIEs) of $\text {CSe}_2^{2+}$ at the MRCI/aug-cc-pVQZ-DK level of theory. VDIEs are obtained as the differences between the energies of the dicationic electronic states and that of $\text {CSe}_2$ ($\hbox {X}^1\Sigma _g^+$), where both molecular species are taken at the $\text {CSe}_2$ ($\hbox {X}^1\Sigma _g^+$) equilibrium geometry. For direct comparison to the experimental spectrum shown in Fig. 1, the theoretical VDIEs are also included in this figure. We note the apparently good agreement between our experimental and theoretical data, with the $\hbox {X}^3\Sigma _g^-$ state at 24.68 eV ionization energy reflecting the doubly-ionized ground state, which is followed by the dicationic 1$^1\Delta _g$ and 1$^1\Sigma _g^+$ states at 25.48 eV and 26.16 eV, and many more, to quite some extent, strongly overlapping excited electronic dicationic states. The energies and assignments of the first 8 states are summarized in Table 1, where assignment of experimental states between 27 and 28 eV is less certain since the peak structure is not apparent. For a more complete presentation of the theoretical VDIEs, the interested reader is referred to the Supplementary Table S1. The spacing of the experimental and theoretical states are in very good agreement with each other, and the offset between the theoretical and experimental energies of about 0.2 eV are within the resolution of the experimental data on the order of ± 0.2 eV.

**Fig. 3:**
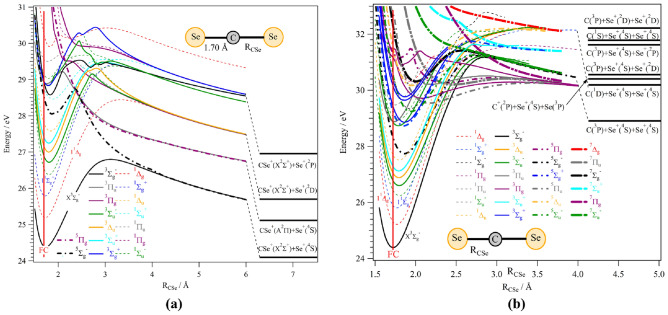
Adiabatic potential energy curves of $\text {CSe}_2^{2+}$ for (**a**) states along the CSe distance whereas the other CSe distance is fixed at 1.70 å, i.e. its value at the ${\hbox {CSe}}_{2}$($\hbox {X}^1\Sigma _g^+$) equilibrium, and (**b**) lengthening both CSe distances symmetrically for linear configurations. In both panel (**a**) and (**b**), the reference energy is that of the ${\hbox {CSe}}_{2}$($\hbox {X}^1\Sigma _g^+$) state at equilibrium, and FC indicates the middle of the Franck–Condon zone relative to the ${\hbox {CSe}}_{2}$($\hbox {X}^1\Sigma _g^+$) state. Dissociation limits were computed at the RCCSD(T)/AUG-CC-PVQZ-DK level and by using values from NIST for atomic excitation energies. Note the different energy scales on the vertical axes.

**Fig. 4:**
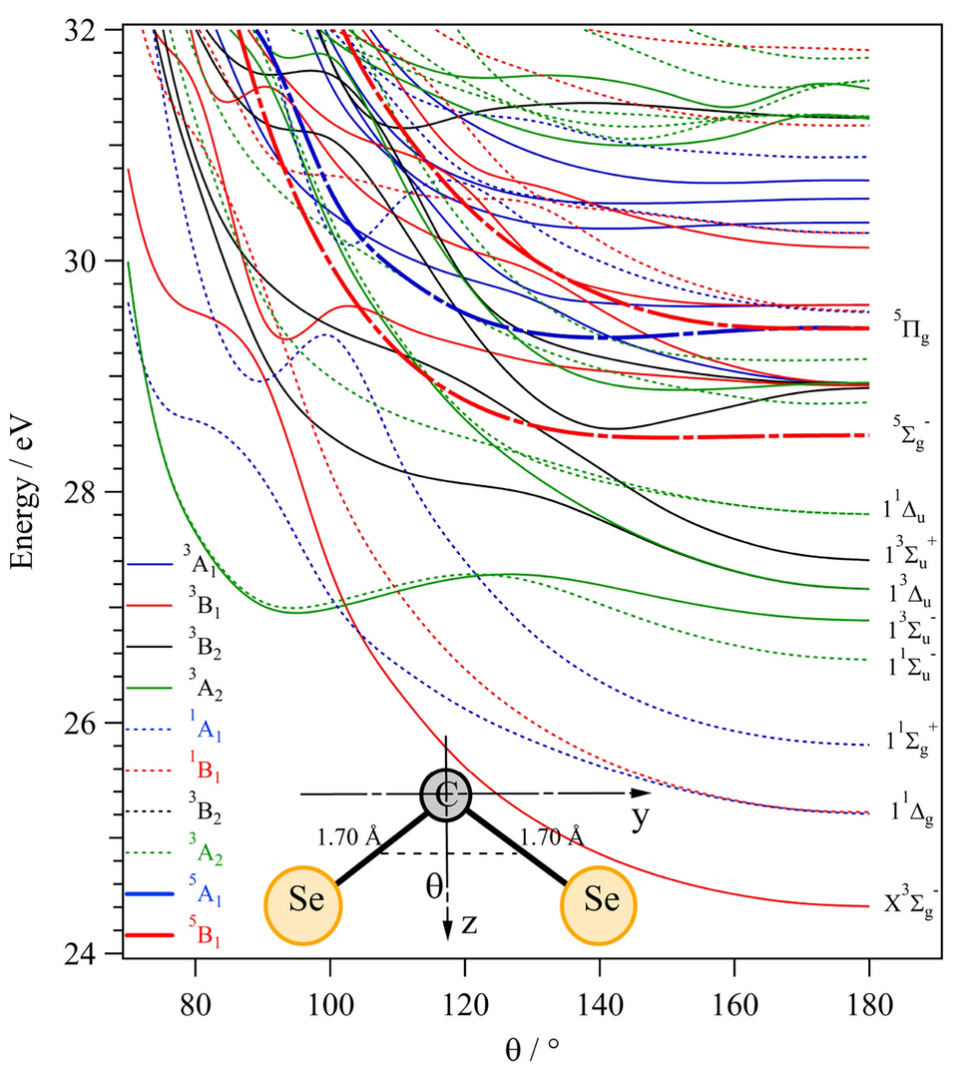
Adiabatic potential energy curves of $\text {CSe}_2^{2+}$ along the in-plane angle $\theta$, where both CSe distances are fixed at 1.70 å, i.e. their value at the ${\hbox {CSe}}_{2}$($\hbox {X}^1\Sigma _g^+$) equilibrium. The reference energy is also that of ${\hbox {CSe}}_{2}$($\hbox {X}^1\Sigma _g^+$) at the same equilibrium. For assignment of the states for bent structures, the molecule is put in the yz-plane. While all states are linear, isomerization may occur on the 1 $^1\Sigma _u^-$ or 1 $^3\Sigma _u^-$ potential energy curves for energies > 29.5 eV.

Let’s concentrate now on the unimolecular fragmentation of $\text {CSe}_2^{2+}$. For this purpose, we show in Fig. 3a one-dimensional cuts of the potential energy surfaces of $\text {CSe}_2^{2+}$ states as a function of the CSe distance where the other CSe distance was fixed at 1.70 å, i.e. its value at the ${\hbox {CSe}}_{2}$($\hbox {X}^1\Sigma ^+_g$) equilibrium, together with dissociation limits for the $\text {CSe}^{+} + \text {Se}^{+}$ channel. We also computed cuts where we lengthened both CSe distances symmetrically (Fig. 3b). These figures reveal several sufficiently deep potential wells, where (meta)stable $\text {CSe}_2^{2+}$ can be found. The metastable states are separated from the corresponding dissociation limits by centrifugal type potential barriers. Similarly, potential energy surface cuts (PECs) for bending the molecule with both CSe distances kept fixed at 1.70 å are given in Fig. 4. This figure shows that most electronic states of this dication possess minima for linear structures. Additional local minima can be seen for bent configurations due to avoided crossings (e.g. between the two lowest $^3A_2$ components). All doubly degenerate states split into two components for bent configurations due to the Renner–Teller effect. Avoided crossings can be observed for collinear configurations. Altogether, we found a high density of electronic states that favours inter-state interactions by vibronic (e.g. at their avoided crossings), spin-orbit (at the crossing between states with different spin multiplicities) and Renner–Teller (for doubly degenerate states) interactions. Consequently, the unimolecular decomposition processes are expected to be very complex, involving multiple steps. The high density of electronic states and the strength of the interactions may even be sufficient to permit a statistical description of the dissociations, such as that embodied in the recent “$\hbox {M}_3$C” computational approach^16^.

In the 22–40 eV photon energy range, we located several dissociation limits for $\text {CSe}_2^{2+}$. At the (R)CCSD(T)/aug-cc-pVQZ-DK level, the lowest charge separation channel $\text {CSe}^+$($\hbox {X}^2\Sigma ^+$) + $\text {Se}^{+}$($^4$S) is computed at 24.10 eV with respect to the ${\hbox {CSe}}_{2}$($\hbox {X}^1\Sigma ^+_g$) minimum energy and after considering Zero Point vibrational Energy (ZPE) corrections. The charge retaining channel (forming $\text {CSe}^{2+}$($\hbox {X}^3\Pi$) + Se ($^3$P) is computed at 34.53 eV. Surprisingly, the lowest Coulomb explosion channels producing $\hbox {Se}^+$ ($^4$S) + $\hbox {Se}^+$($^4$S) + C($^3$P) or $\hbox {Se}^+$ ($^4$S) + Se($^3$) + $\hbox {C}^+$($^2$P) are lying at relatively low energies (at 28.92 eV and 30.59 eV, respectively), and thus distinctly lower than the charge retaining channel.

We calculated a relatively low thermochemical threshold for the ${\hbox {CSe}}_{2}$ $\rightarrow \hbox {Se}_2^+$($\hbox {X}^2\Pi _g$) + $\hbox {C}^+$($^2$P) dissociation of 26.53 eV. We note that this channel requires bond rearrangement of $\text {CSe}_2^{2+}$ from linear to bent structures. Figure 4 shows that such arrangements cannot occur for energies below 27.5 eV since one needs to populate at least the bent minimum of the 1$^1\Sigma ^-_u$ or of the 1$^3\Sigma ^-_u$ state or of those of the upper states. Indeed, the three lowest electronic states of this dication possess unique minima for linear configurations.

As can be seen from Fig. 3a, theory suggests 26.8 eV as the appearance energy (AE) for the $\text {CSe}^{+} + \text {Se}^{+}$ channel, corresponding to the top of the potential barrier of $\text {CSe}_2^{2+}$($\hbox {X}^3\Sigma _g^-$). This computed AE is in line with our experimental observations of the appearance of these products at 26.4 eV. The yield of $\text {CSe}^{+} + \text {Se}^{+}$ continues to dominate the dissociations up to at least 30 eV double ionization energy (DIE) where the spectrum has two more distinct features, one broad peak at around 29.2 and one peak at 31.5 eV. Since these peaks overlap, the AEs for the underlying processes are likely to be somewhat lower than the visible onsets in the spectrum, 28.2 and 30.5 eV, respectively. From the better resolved uppermost electron-only spectrum in Fig. 2 it is evident that at least the first feature consists of several peaks. To identify the internal energies (i.e. electronic and/or vibrational states) of the fragments from dissociation at the DIEs of these two features, experimental AE and KER can be compared with the theoretical results. Several different fragmentation channels are possible according to the PECs and dissociation limits presented in Fig. 3a. Experimental KER values can be estimated from the FWHM of the corresponding ion peaks, a method which may slightly underestimate the actual KERs^17^. Determining the KER separately for the three DIE peaks in the $\text {CSe}^{+} + \text {Se}^{+}$ channel requires four-fold coincidences, and selection on ideally a single Se isotope. This significantly reduces the statistics, making KER determination rather difficult, but from the widths of the ion peaks it is evident that the KER increases for higher DIE. The difference in KER implied from the peak width difference is about 1 eV over the range of DIE from 26.4 to 32.0 eV. A global estimate of the KER from two-fold ion-ion coincidences is 3.2 ± 0.2 eV, arising from all electronic states in the $\text {CSe}^{+} + \text {Se}^{+}$ channel excited at 40.81 eV. This value probably reflects a mean or a dominant contribution, but because the peak width depends on the square root of the energy release, a wide distribution of KERs is not excluded. We also note that the theoretical KERs exclude any vibrational excitation in the $\hbox {CSe}^+$ fragment and so are upper limits. For instance, closer inspection of Fig. 3b, which show PECs for the lengthening of the C-Se distance, suggests that the vibrational excitation energy of $\text {CSe}^+$ could be several eV high before dissociation of the diatomic. Some internal vibrational energy for the $\text {CSe}^+$ fragment is reasonable, since the removal of two electrons from $\pi _\text {u}$ probably will lengthen both CSe bonds. This may result in a longer bond for the $\text {CSe}^+$ fragment compared to its vibrational ground state, and so it becomes vibrationally excited.

Comparison of experimental KER and AE to the possible fragmentation channels in Fig. 3a, allows us to identify the final state of the first DIE peak as $\text {CSe}^+$ ($\hbox {X}^2\Sigma ^+$) + Se+ ($^4$S), with theoretically predicted AE of 26.80 eV and a KER of 2.70 eV. Here, it is clear that fragmentation takes place on the dicationic ground state potential which correlates to the $\text {CSe}^+$ ($\hbox {X}^2\Sigma ^+$) + Se^+^ ($^4$S) dissociation limit. The second theoretical AE of 28.60 eV corresponds to a multi-step process, where we populate first the $\text {CSe}_2^{2+}$ electronic states located in the 26.8–28.8 eV energy range, that convert later by spin-orbit interaction to the long range repulsive part of 1$^5\Sigma ^-_g$ (leading to $\text {CSe}^+$ ($\hbox {X}^2\Sigma ^+$) + Se+ ($^4$S) with a KER of ca. 4.5 eV) or to that of 1$^3\Pi _u$ /1$^5\Pi _g$ (leading to $\hbox {CSe}^+$ $(A^2\Pi$) + $\hbox {Se}^+$($^4$S) with a KER of ca. 3.4 eV). The higher theoretical KER is quite far from the measured mean value and, as mentioned before, we cannot exclude any vibrational excitation in, for instance, the ground state of the $\hbox {CSe}^+$ fragment. Furthermore, we cannot exclude the possibility that $\hbox {CSe}^+$ by this process is formed in the A-state instead of the X-state. For energies > 29.2 eV, a third mechanism is possible, which would lead to the first dissociation limit *via* 1$^5\Sigma ^-_g$, the second dissociation limit *via* 1$^3\Pi _u$ /1$^5\Pi _g$ or to the third dissociation limit (i.e. $\hbox {CSe}^+$ ($\hbox {X}^2\Sigma ^+$) + Se+ ($^2$D)). Closer inspection reveals that the two first pathways are associated with large KERs while only the third one has a theoretical KER of about 3.5 eV close to the experimental mean value. This suggests that the $\hbox {Se}^+$ ions may be produced in the $^2$D state and not the ground $^4$S state. The production of such electronically excited fragments is not surprising in dissociation of a triatomic molecule, where the overall density of states can hardly be sufficient to enforce statistical equilibrium.

Figure 3b shows MRCI/aug-cc-pVQZ-DK potential energy curves of $\text {CSe}_2^{2+}$ states while lengthening both CSe distances symmetrically for linear configurations, together with dissociation limits of the ${\hbox {Se}^{+} + \hbox {C}^{+} + \hbox {Se}}$ and ${\hbox {Se}^{+} + \hbox {Se}^{+} + \hbox {C}}$ channels, where the reference energy is that of the ${\hbox {CSe}}_{2}$($\hbox {X}^1\Sigma ^+_g$) state at equilibrium, and FC marks the middle of the Franck–Condon zone relative to the ${\hbox {CSe}}_{2}$($\hbox {X}^1\Sigma ^+_g$) state. The experimental findings show an AE for the $\text {Se}^{+} +\text { Se}^+$ channel of 32.2 eV, with a KER of 1.2 eV estimated from the ion-ion peak width, while from theory, the AE lies between 31-32 eV, with a calculated KER of 1-2 eV; the uncertainty of these two calculated values is due to the presence of many different states that may contribute. With somewhat higher AE, above 34 eV, the ${\hbox {C}}^{+} + \hbox {Se}^{+}$ fragmentation channel overlaps with the $\text {Se}^{+} +\text { Se}^+$ channel at high DIE. This higher AE is probable for the $\text {C}^{+} + \hbox {Se}^{+}$ channel, since the ionization potential for C is higher than Se, while theory predicts 30.6 eV. As a result of the numerous states involved, it is difficult to determine more specifically the path of this dissociation.

An important observation in the ion-ion coincidence map of doubly-ionized ${\hbox {CSe}}_{2}$ is that the slope for the coincidence island of $\text {Se}^{+} + \text {C}^+$ channel is very steep, about −4 ± 1, showing that the $\hbox {C}^+$ ion is a secondary product from sequential decay of $\hbox {CSe}^+$. In contrast, the $\text {Se}^{+} +\text { Se}^+$ island has a slope equal to $-1$, suggesting a simple, possibly instantaneous explosion of the original symmetrical dication. If the sequential decay of $\hbox {CSe}^+$ takes place after completely leaving the Coulomb zone and losing all initial angular alignment relative to the $\hbox {Se}^+$ ion, the slope of the island would be $-7.7$ (from linear momentum conservation and the masses of the fragments). The experimental slope shows that the sequential reaction is ${\text {CSe}_{2}^{2+} \rightarrow \hbox {Se}^{+} + \hbox {CSe}^{+} \rightarrow \text {Se}^{+} + \hbox {C}^{+} + \text {Se}}$, and not secondary decay of $\hbox {Se}_2^+$ as in that (unlikely) case the slope would be $-0.5$.

We note that from a thermodynamical point of view and according to our theoretical investigations, the exotic double ionization channel $\text {CSe}_2$ ($\hbox {X}^1\Sigma ^+_g$) $\rightarrow$ $\hbox {Se}_2^+$ (X $^2\Pi _g$) + $\hbox {C}^+$ ($^2$P) is expected to be energetically accessible from 26.53 eV + some KER, while no experimental evidence for this channel was found within the energy range investigated. By having a closer look at the PECs shown in Fig. 4, it is reasonable to say that all the states are linear in the Franck–Condon region accessible from the ${\hbox {CSe}}_{2}$ ($\hbox {X}^1\Sigma ^+_g$) state, which implies that, upon double ionization, bending or isomerisation to form $\hbox {Se}_2^+$ is unlikely to occur. Furthermore, a possible explanation for not observing the formation of $\text {Se}_2^+$ from double ionization could be that Coulomb explosion of $\text {CSe}_2^{2+}$ takes place already at an unusually low energy, while for other analogue systems the dimer production is observed instead. This is especially the case of inner-shell ionization of ${\hbox {CO}}_{2}$ and of valence double ionization of $\text {SO}_2$, for which the channel producing $\text {O}_2^+$ has been reported in the literature^3,18,19^, although studies on the other analogous system, ${\hbox {CS}}_{2}$ by Lablanquie et al.^20^ does not suggest the production of the $\text {S}_2^+$ dimer. Apart from that, no metastable $\text {CSe}_2^{2+}$ has been detected, while metastable $\text {CS}_2^{2+}$ and $\text {CO}_2^{2+}$ species are known in the literature^20,21^.

The charge retaining double ionization channel $\text {CSe}_2$ ($^1\Sigma ^+_g$) $->$ $\hbox {CSe}^{2+}$ (X $^3\Pi$) + Se($^3$P) is predicted by our calculations to occur at 34.53 eV and onwards. Although seen in the ion spectra, from the present multi-dimensional data sets, this channel is statistically not sufficiently significant to deduce any AE or DIE values. We believe that the weak observation of the charge retaining channel, although it is thermodynamically allowed, is due to the presence of low lying states leading to Coulombic explosion.

## Conclusions

By utilising versatile multi-particle coincidence spectroscopy in combination with advanced quantum chemical calculations, we have characterized the electronic structure and the fate of $\text {CSe}_2^{2+}$ including its fragmentation channels, with a very good agreement between experiment and theory. The lowest VDIE of ${\hbox {CSe}}_{2}$ was determined to be 24.68 ± 0.20 eV, and the fragmentation channel producing $\text {CSe}^{+} + \text {Se}^{+}$ is found to be the strongest with an experimental appearance energy of 26.4 eV. In searching for more exotic fragmentation channels, our findings indicate that doubly-ionized ${\hbox {CSe}}_{2}$ is surprisingly unlike closely related molecules such as $\text {SO}_2$ and ${\hbox {CO}}_{2}$.

**Table 1:** Numerical comparison of experimental and theoretical Vertical Double Ionization Energies (VDIEs) of ${\hbox {CSe}}_{2}$. For the experimental VDIE, the uncertainty is ± 0.2 eV.

State	Theoretical VDIE (eV)	Experimental VDIE (eV)
X $^3\Sigma _g^-$	24.41	24.68
1 $^1\Delta _g$	25.22	25.48
1 $^1\Sigma _g^+$	25.82	26.16
1 $^1\Sigma _u^-$	26.55	26.80
1 $^3\Sigma _u^-$	26.89	27.20
1 $^3\Delta _u$	27.16	27.44
1 $^3\Sigma _u^+$	27.41	27.76
1 $^1\Delta _u$	27.81	27.98

With regard to desirable future investigations, more highly resolved electron spectra of the ${\hbox {CSe}}_{2}$ dication are likely to provide an in-depth understanding of its expected vibrational structure and on the possible perturbations between its electronic and vibrational states. Such spectra are possibly within reach using a related experimental set-up with a substantially higher resolving power due to a much longer electron flight tube employed (cf. Refs.^22,23^), which is in the process of being refurbished. Also, such spectra could be computed using the electronic methodologies described in Ref.^24^ complemented by nuclear motion treatments as done for $\hbox {CO}_2^{2+}$, $\hbox {OCS}^{2+}$ and $\hbox {CS}_2^{2+}$ (cf. Refs.^25,26^). Nevertheless, one needs to extent these methodologies to consider a substantially larger number of electronic states for describing the $\hbox {CSe}_2^{2+}$ electronic states located above 32 eV with respect to the $\hbox {CSe}_2$ ground state.

## Methods

### Experimental details

Multi-particle coincidence experiments were carried out in our laboratory at the University of Gothenburg, utilising our versatile magnetic bottle time-of-flight photoelectron–photoelectron–photoion–photoion coincidence (TOF-PEPEPIPICO) spectrometer set-up, that has been described in detail before^1^. In its basic electron-only configuration, this set-up allows efficient measurement of energy-resolved electron pair spectra with a nominal resolving power of *E*/$\Delta E$ = 50 and a collection-detection efficiency of about 50%. In its advanced configuration, electron pair spectra are measured with a reduced nominal resolving power of *E*/$\Delta E$ = 20, but with the same aforementioned efficiency and in correlation with singly or doubly-charged ions which are differentiated based on their mass/charge ratio. As ionizing radiation source, a pulsed helium gas discharge lamp was employed which provides intense atomic emission lines of HeI$\alpha$ and HeII$\alpha$ at 21.2 eV and 40.8 eV, respectively. The target molecules enter the light-matter interaction region through a hollow needle forming an effusive jet that intersects the wavelength-selected ionizing radiation. For double ionization measurements HeII$\alpha$ radiation was required. A weak electric field ($\sim$1 V) is applied across the ionization region to accelerate near-zero energy electrons into the 2.2 m long electron flight tube. Using a ring magnet which provides a few hundred mT magnetic collimating field, instead of the usual, about 1 T strong solid magnet, allows us to collect the electrons and ions in opposing directions. The ions are extracted by a strong, pulsed electrical field and pass through an accelerating electric field, in total of about 600 V, before passing through the ring magnet into a time-of-flight mass spectrometer of about 0.12 m length. The two-field configuration optimized for time focusing conditions following the original concept of Wiley–McLaren^27^ is applied. The strength of the extraction field and the associated acceleration field determines the mass resolution and the flight time width of signals for fragment ions formed with more than thermal kinetic energy. To ensure that all the relevant electrons have left the interaction region before the ion extraction field is applied, a delay of about 50 ns relative to the arrival of the ionizing radiation pulse is used. Typical mass resolution for ions is about 1 atomic unit at m/z 100, with a collection-detection efficiency for ions of about 20%.

Carbon diselenide was synthetized through the reaction of selenium powder with dichloromethane (DCM) vapour at $550^\circ$C:

$$\begin{aligned} 2 \text {Se }+ \text {CH}_2\text {Cl}_2 \xrightarrow {550^\circ \text {C}} \text {CSe}_2 + 2 \text {HCl} \end{aligned}$$

Since $\text {CSe}_2$ easily reacts with air to form a series of highly toxic and rather noxious compounds, all synthesis work was conducted in a ventilated fume hood. Selenium powder and DCM were purchased commercially from Sigma Aldrich. A satisfactory synthesis apparatus consisted of a Pyrex three-necked round-bottomed reaction flask, wrapped with heating wires connected to a power supply and insulated with fiber glass sheathes. Selenium powder was poured into the flask, which was placed in a Pyrex glass container filled with vermiculite, to insulate the reaction vessel and reduce heating loss. DCM vapour, provided by a vessel containing the liquid and maintained at a fixed temperature, was delivered from above to the surface of molten selenium in a stream of argon. The flask was connected to a Liebig condenser and the product was collected in a trap maintained at $-78^\circ$C by using an ethanol/liquid nitrogen slush bath. The final product consisted of a bright yellow liquid with a pungent and foul odor, with DCM as the main impurity. The sample was purified through several vacuum pumping cycles at $-78^\circ$C ($\hbox {CSe}_{2\text {mp}}$ = $-43.7^\circ$C, $\hbox {DCM}_{\text {mp}}$ = $-96.7^\circ$C). Even pure ${\hbox {CSe}}_{2}$ is susceptible to light and will spontaneously decompose over a few days, going from a bright yellow liquid to a black solution with some precipitation occurring. During the experiments, the sample purity was checked frequently by on-line ion time-of-flight spectroscopy.

### Computational details

Previous theoretical works regarding Se containing molecular species pointed out the importance of considering a large amount of electron correlation and of relativistic effects while computing their energetics and spectroscopic properties^28–30^. Here, a first screening of the electronic wavefunctions of $\hbox {CSe}_2^{2+}$ states confirmed the need of large scale computations to correctly predict the pattern of the lowest electronic states of this dication. Indeed, we found that the wavefunctions of these electronic states exhibit strong multiconfigurational character. Also, the dicationic potentials are found to be very flat along the bending coordinates, resulting in low frequency modes, hardly described by low levels of theory, in particular for such molecular systems where we identified Hartree-Fock (HF) instability (i.e. symmetry breaking) problems to be solved. In addition, we needed to consider relativistic effects because of Se. We determined that Coupled Cluster methods lead to unphysical equilibrium structures and energetics in the molecular region, since they are monoconfigurational and rely on a (unique) HF determinant which is useless due to HF instability. Therefore, we performed computations using the internally contracted Multi-Reference Configuration-Interaction (MRCI)^31–33^ method on top of the Complete Active Space Self Consistent Field (CASSCF)^34,35^ technique as implemented in MOLPRO 2015^36^. The computations were performed in the $\hbox {C}_{2v}$ point group, where the $\hbox {B}_1$ and $\hbox {B}_2$ representations were treated as equivalent.

At the CASSCF level, we considered all valence electrons and all valence molecular orbitals as active. Then, the MRCI active space was constructed after including all configurations in the CI expansion of the CASSCF wavefunctions. This results in > 3.8 $\times$ 10$^9$, > 7.4 $\times$ 10$^9$ and > 5.1 $\times$ 10$^9$ Configurations State Functions (CSFs), per $\hbox {C}_{2v}$ symmetry, while computing the singlets, triplets and quintets states of $\text {CSe}_2^{2+}$, respectively. In fact, this dication exhibits a high density of electronic states (see below) making these electronic structure computations quite challenging to converge.

To deduce the adiabatic ionization energy of $\text {CSe}_2$ and to locate the dissociation limits of $\hbox {CSe}_2^{2+}$, we had to optimize the geometries of $\text {CSe}_2$ and those of its neutral and ionic fragments, where we used the Coupled Clusters (R)CCSD(T)-DK method as implemented in MOLPRO 2015, since MRCI is not size consistent and thus cannot be used for those purposes. Prior to that, we checked the monoconfigurational nature of all atoms and diatomic species that we considered (both neutral, singly and doubly charged). We also optimized the neutral ground state of ${\hbox {CSe}}_{2}$ at this level of theory. For all molecular species, we computed the harmonic frequencies to confirm that they correspond to minima (all > 0 frequencies) and to deduce the corresponding Zero Point vibrational Energies (ZPEs). ZPEs are later used in deriving the adiabatic ionization and dissociation energies.

For all electronic structure computations, the C and Se atoms were described using the all-electron Douglas-Kroll Valence Quadruple Zeta + Polarization (aug-cc-pVQZ-DK) basis set^37^.

## Supplementary Information

Supplementary Information.

## Data Availability

The data sets generated during and/or analysed during the current study are available from the corresponding authors on reasonable request.
