# Supplementary material for: Characterization of the electronic structure and fate of doubly ionized carbon diselenide
Source: Sci Rep. 2025 Feb 24;15:6664. doi: 10.1038/s41598-025-90637-5 (PMC11850846; doi:10.1038/s41598-025-90637-5)
Supplement: Supplementary file 1 — Supplementary Information. [file 41598_2025_90637_MOESM1_ESM.pdf]

Supplementary Materials: Characterization of the electronic structure and fate of doubly ionized carbon diselenide

Marco Parriani[1,2,+], Emelie Olsson[2,+], Veronica Daver Ideböhn[2], Måns Wallner[2], Richard J. Squibb[2], Gunnar Nyman[3], Stefano Falcinelli[1], John H.D. Eland[4], Majdi Hochlaf[5,*], and Raimund Feifel[2,]**

[1]University of Perugia, Department of Civil and Environmental Engineering, Via G. Duranti 93, 06125 Perugia, Italy
[2]University of Gothenburg, Department of Physics, Origovägen 6B, 412 58 Gothenburg, Sweden
[3]University of Gothenburg, Department of Chemistry and Molecular Biology, Box 462, 405 30 Gothenburg, Sweden
[4]Oxford University, Department of Chemistry, Physical and Theoretical Chemistry Laboratory, South Parks Road, Oxford OX1 3QZ, United Kingdom
[5]Université Gustave Eiffel, COSYS/IMSE, 5 Bd Descartes, 77454 Champs sur Marne, France
[+]these authors contributed equally to this work
[*]majdi.hochlaf@univ-eiffel.fr
[**]raimund.feifel@physics.gu.se

Table S1. Comparison of theoretical and experimental vertical double ionization energies of CSe_2, with tentative assignments. The resolution of the experimental data is on the order of ± 0.02 eV, whereas the uncertainty of the absolute experimental double ionization energy is approximately ± 0.2 eV.

State	Theoretical VDIE (eV)	Experimental VDIE (eV)
X $^3\Sigma_g^-$	24.41	24.68
1 $^1\Delta_g$	25.22	25.48
1 $^1\Sigma_g^+$	25.82	26.16
1 $^1\Sigma_u^-$	26.55	26.80
1 $^3\Sigma_u^-$	26.89	27.20
1 $^3\Delta_u$	27.16	27.44
1 $^3\Sigma_u^+$	27.41	27.76
1 $^1\Delta_u$	27.81	27.98
1 $^1\Sigma_u^+$	28.75	
2 $^3\Delta_u$	28.91	29.12
2 $^3\Sigma_g^-$	28.92	
1 $^3\Pi_u$	28.93	
2 $^1\Sigma_u^-$	29.17	
1 $^1\Pi_u$	29.56	29.84
1 $^3\Delta_g$	29.62	
2 $^1\Delta_g$	30.25	
1 $^3\Sigma_g^+$	30.33	
2 $^3\Sigma_g^+$	30.54	
3 $^1\Delta_g$	31.19	
1 $^3\Pi_g$	31.22	
1 $^1\Pi_g$	31.27	
2 $^3\Pi_g$	31.48	
2 $^1\Delta_u$	31.57	31.72
3 $^3\Pi_g$	32.34	
